# Rapid Hepatitis C Testing Among Persons at Increased Risk for Infection — Wisconsin, 2012–2013

**Published:** 2014-04-11

**Authors:** Lauren J. Stockman, Sheila M. Guilfoyle, Andrea L. Benoit, James M. Vergeront, Jeffrey P. Davis

**Affiliations:** 1 Wisconsin Division of Public Health

An estimated 3.2 million persons in the United States have chronic infection with hepatitis C virus (HCV) (1). Most new HCV transmissions occur among persons who inject drugs (2), often within the first few years of their injection drug use (3). During 2003–2012, reports of HCV infection increased from 15 to 54 cases per 100,000 among persons aged <30 years in Wisconsin, and 58% of persons in this age group with acute HCV infection reported injecting drugs (Wisconsin Division of Public Health, unpublished data, 2013). To increase detection of HCV infection, the Wisconsin Division of Public Health (WDPH) piloted a program during October 2012–October 2013 that offered rapid HCV testing to clients of four agencies providing outreach testing for HCV and human immunodeficiency virus infection, syringe exchange, counseling, and other harm reduction services to persons with drug dependence. During that period, 1,255 persons were tested using a rapid HCV test, and 246 (20%) of the results were positive. Most (72%) of the infections had not been reported to WDPH. A blood specimen for further testing was collected from 192 (78%) participants with positive HCV test results; among these participants, 183 were tested for HCV RNA using reverse transcription–polymerase chain reaction (RT-PCR), and these results were positive for 128 (70%) participants, indicating active infection. Use of the rapid HCV test detected previously unreported HCV infections and raised awareness of HCV. Persons identified with active HCV infection should be referred to medical care and counseled on ways to prevent HCV transmission to others.

A new cohort of young injection drug users acquiring HCV infection has been recognized nationwide, notably in suburban and rural areas (4). Most persons infected with HCV are unaware of their infection because it has few, if any, symptoms.* CDC and the United States Preventative Service Task Force recommend that persons who inject drugs receive HCV enzyme immunoassay (EIA) and nucleic acid testing to diagnose current HCV infections (5,6). Conventional EIA testing requires laboratory equipment, a trained phlebotomist, and long turnaround times to process. However, rapid point-of-care screening tests for HCV antibody using finger-stick capillary blood allow screening to be expanded outside of a clinical setting (7,8).

WDPH, in partnership with community organizations, supports outreach and overdose prevention services for persons with drug dependence. Four outreach agencies providing these services currently use rapid HIV testing technology and have staff members trained to collect blood specimens for PCR testing. Staff members at each agency were provided with OraQuick rapid HCV test kits (OraSure Technologies). Confirmatory tests using EIA and PCR were conducted at the Wisconsin State Laboratory of Hygiene.

Clients who used services from these agencies during October 2012–October 2013 were offered the OraQuick test and were interviewed to collect information regarding demographic characteristics and risk behaviors. Participants whose OraQuick test results were positive had blood specimens obtained by venipuncture and were asked to return to the outreach site for confirmatory test results. Reactive tests, and confirmatory results, when available, were reported to WDPH. Data from the Wisconsin Electronic Disease Surveillance System were analyzed to determine whether HCV infections detected during this pilot program had been reported previously to WDPH from a laboratory or local health department.

During the pilot program, rapid HCV tests were performed on blood specimens from the 1,255 participants, and results for 246 (20%) were positive. Most participants reported either that they had not been tested previously for HCV (53%) or they did not remember being tested for HCV (10%). Most (72%) of the infections detected during the pilot period were not recorded in the Wisconsin Electronic Disease Surveillance System, indicating they were newly detected HCV infections and not previously reported to WDPH.

Of the 1,255 persons who received rapid HCV tests, the median age was 28 years (range = 17–68 years), and 732 (59%) were male (Table). A total of 965 (78%) participants were non-Hispanic white, 81 (7%) were non-Hispanic black, 79 (6%) were non-Hispanic American Indian, and 18 (1%) were of other, mixed, or nonspecified race. A total of 97 (8%) were of Hispanic or Latino ethnicity. Compared with 2012 state data on HCV cases reported to WDPH, the 246 pilot program participants with HCV infection were younger (49% aged <30 years compared with 24%), more likely to be non-Hispanic white (78% compared with 63%), and less likely to be non-Hispanic black (5% compared with 13%) (Table).

The most common risk behavior or exposure reported by participants was injection drug use, reported by 1,033 (82%) of the 1,255 participants. A total of 868 (69%) reported injecting drugs within 6 months of testing (Figure); among these participants, the average number of persons with whom they reported injecting drugs was 5.4 (range = zero to 100). Among the participants, 825 (66%) reported sharing drug injection equipment, and 531 (42%) reported sharing equipment within 6 months of testing.

A blood specimen for confirmatory testing was collected from 192 (78%) of the 246 participants with positive results from rapid HCV tests. The 54 participants without a blood specimen either refused venipuncture or agency staff members could not access a vein and referred them to a clinic for HCV diagnostic testing. Among participants with a blood specimen, 190 of the 192 were tested for HCV antibody using EIA, and 100% of the results were positive, indicating no false-positive results from the rapid HCV test. A total of 183 of the 192 were tested for HCV RNA using RT-PCR, and 128 (70%) had a positive result, indicating a high proportion of participants with active infection.

## Discussion

As a tool for enhanced surveillance in Wisconsin, use of the rapid HCV test facilitated screening at four agencies where predominantly young persons at increased risk for HCV infection receive outreach testing, syringe exchange, and other harm reduction services. Most persons who inject drugs acquire HCV infection during their first years of injecting, and sharing of injection equipment has been found to be the most important behavior associated with infection (3,9). The prevalence of HCV infection among program participants was 20%. Results of previous studies have demonstrated higher prevalence of HCV infection among persons who inject drugs in the United States (range = 35%–65%) (9). However, participants in this program were tested within agencies offering harm reduction services and, therefore, might exhibit safer injection practices or more preventive behaviors compared with other persons who inject drugs.

A majority (70%) of the HCV infections detected during this program were active, viremic infections. This finding, coupled with self-reported behaviors of recent and continued sharing of injection equipment despite participation in a syringe exchange service, is concerning. These data suggest that prevention messages that emphasize the risk for HCV transmission during the sharing of any injection equipment (not only syringes) are highly important. All-oral therapeutic regimens with shorter duration and fewer adverse effects are now available to cure HCV infection and reduce the risk for transmission. Efforts to link persons with viremia to medical care are critical to limit the spread and impact of hepatitis C in Wisconsin.

Although use of the rapid HCV test provides a result at the point-of-visit, additional tests are needed to differentiate past infections from current infections. Therefore, HCV antibody surveillance strategies also should include the collection of blood specimens for RNA diagnostic testing. In the current program, venous blood specimens were obtained from 78% of participants with positive results from rapid HCV tests. The number of specimens collected to confirm current HCV infection might be increased, where practical, with additional training in venipuncture at each agency and enhancement of messages informing clients of the importance of HCV testing and knowledge of the test results.

The findings in this report are subject to at least three limitations. First, although rapid HCV tests were readily available, they were not accepted by all clients at the four agency sites, and data regarding rates of refusal of the rapid tests were not collected systematically. Second, efforts were made by each agency to refer clients with positive HCV test results to medical services; however, data regarding linkage to treatment of acute HCV infection were not collected systematically. Such data are essential to help determine where efforts to improve access to treatment might be needed. Finally, populations served by the four agencies might not be representative of the general Wisconsin population, which might account for all or part of the demographic differences between the HCV-positive population in Wisconsin and the persons with positive HCV test results in this pilot program.

During this pilot program, a rapid HCV test was used to increase HCV awareness and diagnose HCV infections within the context of supportive and accessible community public health programs. This partnership of state public health and community organizations can play an important role in efforts to decrease HCV infections among young persons who inject drugs. Recent evidence suggests that availability of HCV detection services in integrated care settings that combine substance abuse treatment and injection safety is most effective at reducing HCV infection among persons who inject drugs (10). The use of rapid HCV tests could be a powerful tool for screening, conveying prevention information, and initiating treatment in this population with a high prevalence of HCV infection.

What is already known on this topic?

Hepatitis C virus (HCV) infection is readily transmitted through injection drug use. A new cohort of young injection drug users acquiring HCV infection has been recognized nationwide, notably in suburban and rural areas.

What is added by this report?

Rapid HCV tests were used to test 1,255 persons at increased risk for HCV infection. Of these, 20% had positive HCV test results, and 72% of the infections had not been reported previously. Blood specimens for confirmatory testing were collected from 78% of the participants with positive test results, and 70% of those specimens tested positive for HCV RNA, indicating a high proportion of participants with active infection.

What are the implications for public health practice?

The use of rapid HCV tests could be a powerful tool for conducting HCV screening, conveying prevention information, and initiating treatment in a population with high prevalence of HCV infection.

## Figures and Tables

**FIGURE:**
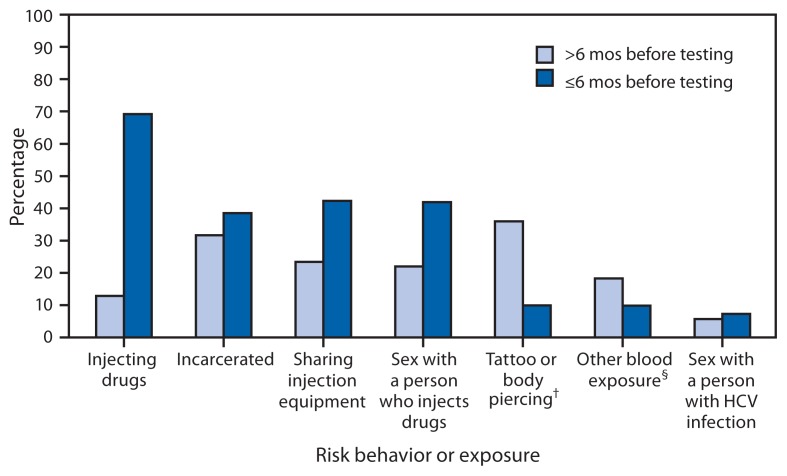
Percentage of participants (N = 1,255) reporting selected risk behaviors or exposures,^*^ by period before rapid hepatitis C virus (HCV) testing during a pilot program — four outreach agencies, Wisconsin, 2012–2013 ^*^Participants could report more than one risk behavior or exposure. ^†^From an unlicensed vendor. ^§^For example, contact with blood during a fight.

**TABLE:** Demographic characteristics of persons who received a rapid hepatitis C virus (HCV) test in a pilot program, compared with 2012 state surveillance data on HCV infection* — Wisconsin, October 2012–October 2013

Characteristic	Received test (N = 1,255)		Positive test result (n = 246)		No. of cases reported by state in 2012 (N = 2,634)		p-value†
		
	No.	(%)	No.	(%)	No.	(%)	
**Sex (n = 1,231)**							0.13
Male	732	(59)	136	(55)	1,585	(60)	
Female	499	(41)	110	(45)	1,049	(40)	
**Age group (yrs) (n = 1,223)**							<0.01
<30	698	(57)	120	(49)	636	(24)	
≥30	525	(43)	126	(51)	1,998	(76)	
**Race/Ethnicity (n = 1,240)**							
White, non-Hispanic	965	(78)	193	(78)	1,665	(63)	<0.01
Hispanic or Latino	97	(8)	23	(9)	138	(5)	<0.01
Black, non-Hispanic	81	(7)	12	(5)	346	(13)	<0.01
American Indian, non-Hispanic	79	(6)	16	(7)	63	(2)	0.02
Other§	18	(1)	2	(1)	59	(2)	0.17

* Available at http://www.dhs.wisconsin.gov/publications/p0/p00440-2012.pdf.

† p≤0.05 indicates statistically significant difference between pilot program results and 2012 Wisconsin HCV surveillance data.

§ Includes Asian, Hawaiian or Pacific Islander, mixed, and unspecified race. Race/ethnicity response was missing from 373 (14%) of the 2,634 Wisconsin surveillance records for 2012.

## Footnotes

* Additional information available at http://www.cdc.gov/hepatitis/hcv/index.htm.
